# A novel surgical scheme for hepatectomy in hepatocellular carcinoma patients with clinically significant portal hypertension

**DOI:** 10.1186/s12885-024-12535-9

**Published:** 2024-06-25

**Authors:** Jia-zhou Ye, Hua-ze Lu, Can Zeng, Guo Lei, Xiao-bo Wang, Jie Chen, Tao Bai, Fei-xiang Wu, Rong-yun Mai, Wei-xing Guo, Le-qun Li

**Affiliations:** 1 Department of Hepatobiliary Surgery, Guangxi Medical University Cancer Hospital, Nanning, 530021 China; 2 Department of Hepatic Suegery VI, Eastern Hepatobiliary Surgery Hospital, Second Military Medical University, 225 Changhai Road, Shanghai, 200438 China

**Keywords:** Hepatocellular carcinoma, Portal hypertension, Post-hepatectomy liver failure, Nomogram, Conditional inference tree

## Abstract

**Objective:**

Clinically significant portal hypertension (CSPH) seriously affects the feasibility and safety of surgical treatment for hepatocellular carcinoma (HCC) patients. The aim of this study was to establish a new surgical scheme defining risk classification of post-hepatectomy liver failure (PHLF) to facilitate the surgical decision-making and identify suitable candidates for individual hepatectomy among HCC patients with CSPH.

**Backgrounds:**

Hepatectomy is the preferred treatment for HCC. Surgeons must maintain a balance between the expected oncological outcomes of HCC removal and short-term risks of severe PHLF and morbidity. CSPH aggravates liver decompensation and increases the risk of severe PHLF thus complicating hepatectomy for HCC.

**Methods:**

Multivariate logistic regression and stochastic forest algorithm were performed, then the independent risk factors of severe PHLF were included in a nomogram to determine the risk of severe PHLF. Further, a conditional inference tree (CTREE) through recursive partitioning analysis validated supplement the misdiagnostic threshold of the nomogram.

**Results:**

This study included 924 patients, of whom 137 patients (14.8%) suffered from mild-CSPH and 66 patients suffered from (7.1%) with severe-CSPH confirmed preoperatively. Our data showed that preoperative prolonged prothrombin time, total bilirubin, indocyanine green retention rate at 15 min, CSPH grade, and standard future liver remnant volume were independent predictors of severe PHLF. By incorporating these factors, the nomogram achieved good prediction performance in assessing severe PHLF risk, and its concordance statistic was 0.891, 0.850 and 0.872 in the training cohort, internal validation cohort and external validation cohort, respectively, and good calibration curves were obtained. Moreover, the calculations of total points of diagnostic errors with 95% CI were concentrated in 110.5 (range 76.9-178.5). It showed a low risk of severe PHLF (2.3%), indicating hepatectomy is feasible when the points fall below 76.9, while the risk of severe PHLF is extremely high (93.8%) and hepatectomy should be rigorously restricted at scores over 178.5. Patients with points within the misdiagnosis threshold were further examined using CTREE according to a hierarchic order of factors represented by the presence of CSPH grade, ICG-R15, and sFLR.

**Conclusion:**

This new surgical scheme established in our study is practical to stratify risk classification in assessing severe PHLF, thereby facilitating surgical decision-making and identifying suitable candidates for individual hepatectomy.

**Supplementary Information:**

The online version contains supplementary material available at 10.1186/s12885-024-12535-9.

## Introduction

Hepatocellular carcinoma (HCC) is the sixth most common cancer and the second leading cause of cancer-related death worldwide. Approximately 90% of HCC cases are associated with advanced hepatic fibrosis or cirrhosis due to hepatocyte damage caused by chronic liver diseases including hepatitis B, hepatitis C, chronic alcohol abuse, diabetes mellitus and obesity-related non-alcoholic steatohepatitis (NASH) [1]. Pathological alterations of liver parenchyma, together with increases in transhepatic perfusion resistance and portal venous pressure (PVP), tend to develop into liver function decompensated, and progress to clinically significant portal hypertension (CSPH), which up to 55% of HCC patients [2].

Hepatectomy is the preferred curative treatment for very early-/early-stage HCC and partial intermediate/advanced HCC with resectable tumors, good general performance status, and well-preserved liver function, yielding a 5-year survival benefit [3]. Nevertheless, post-hepatectomy liver failure (PHLF), which is a serious complication, predominantly contributes to postoperative mortality [4]. In particular, CSPH aggravates liver dysfunction, and increases the risk of PHLF, so complicates surgical treatment for HCC [5]. In this regard, the Barcelona Clinic Liver Cancer (BCLC) diagnosis and treatment algorithm considered CSPH as an absolute contradiction for hepatectomy for HCC when it was firstly proposed in 1999 [6]. The European Association for the Study of Liver Disease (EASL) [7] in 2001 and the American Association for the Study of Liver Disease (AASLD) [8] in 2005 advocated that hepatectomy was merely applied for single tumor and Child-Pugh A liver function with total bilirubin (T-Bil) < 1 mg/dl and absence of CSPH. Instead, liver transplantation (LT) was recommended for HCC patients with CSPH and poorly preserved liver function. Nonetheless, the use of LT has been extremely limited in clinical practice because of a shortage of liver donors^1^, high price and liver graft dysfunction due to chronic immune mediate injury [9].

**Fig. 1:**
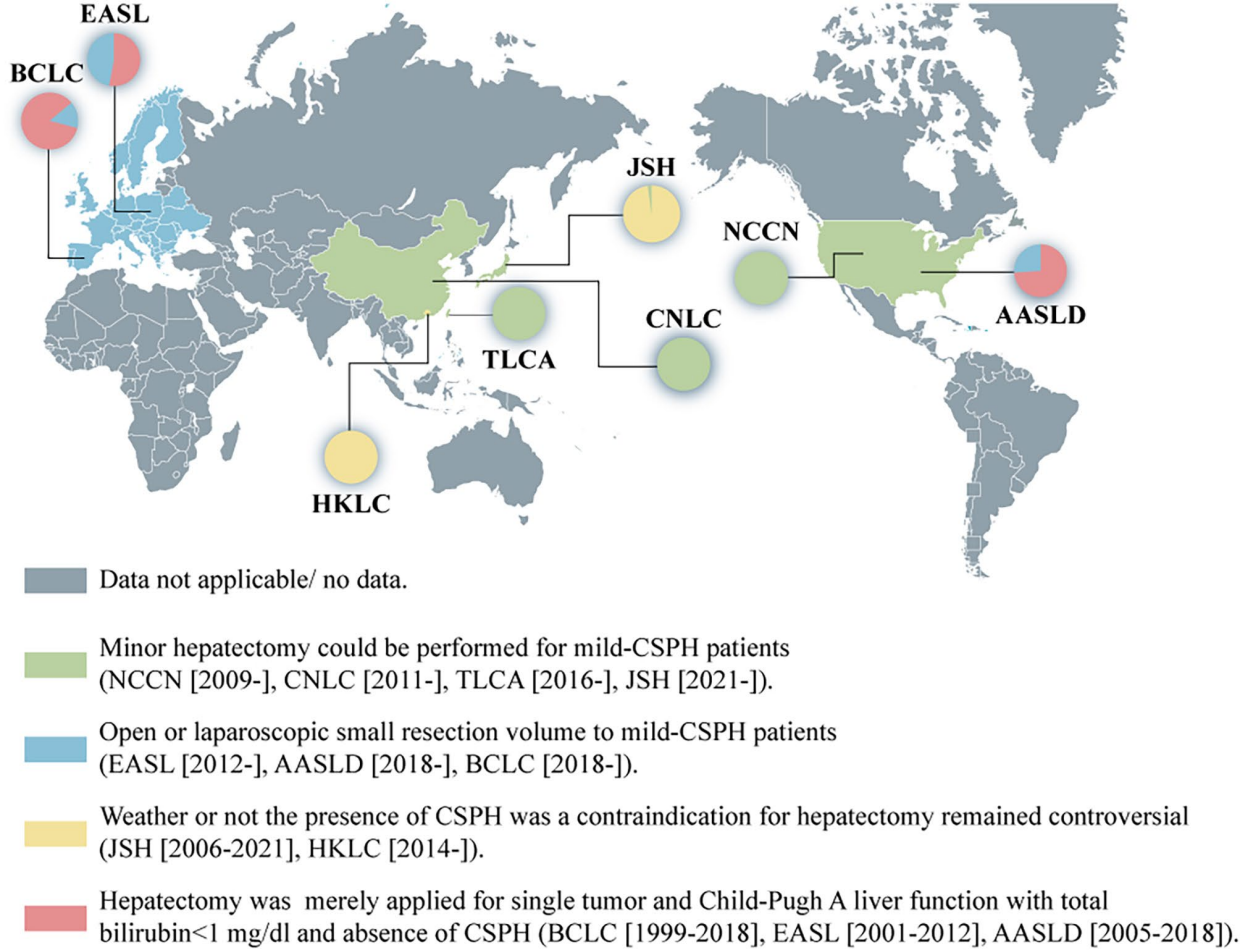
Development of indications for hepatic resection in Western and Asia-Pacific HCC guidelines. *Abbreviations*: BCLC, Barcelona Clinic Liver Cancer; AASLD, American Association for the Study of Liver Disease; EASL, European Association for the Study of Liver Disease; NCCN, National Comprehensive Cancer Network; TLCA, Taiwan Liver Cancer Association; CNLC, China Liver Cancer Staging; JSH, Japan Society of Hepatology; HKLC, Hong Kong Liver Cancer; HCC, hepatocellular carcinoma; CSPH, clinically significant portal hypertension; PVTT, protal vein tumor thrombus

Over the past few decades, with improvement of surgical techniques and peri-operative care, the morbidity of post-operative complications and mortality have decreased greatly. Restriction of CSPH on hepatectomy have been challenged based on the fact that hepatectomy for patients with preserved liver function and moderate CSPH evidently yield competitive survival outcomes in comparison to patients without CSPH [10–12]. The National Comprehensive Cancer Network (NCCN) in 2009 [13] updated their proposal to expand minor hepatectomy to patients with CSPH and well-preserved liver function. And EASL in 2012 [14], AASLD in 2018 [15], as well as BCLC in 2018 [16] accepted this criteria that either open or laparoscopic resection for small resection volume to patients with mild CSPH. In Asian-Pacific regions, the China Liver Cancer (CNLC) since 2011 [17] considered slightly elevated bilirubin or portal hypertension as not a definite contradiction for surgical resection. Further, in 2019, CNLC evidently supported EASL and revised the guideline to expand the criteria for hepatectomy based on stratification of Child-Pugh class liver function and multiparametric evaluations: for compensated Child-Pugh class A with a model for end-stage liver disease (MELD) score < 10, an acceptable grade of portal hypertension matched with a suitable amount of remaining parenchyma: for Child-Pugh class B with moderate portal hypertension the possibility to undergo a laparoscopic or robotic assisted/minimally invasive approach. Taiwan Liver Cancer Association (TLCA) in 2016 [18], Japan Society of Hepatology (JSH) in 2021 [19], updated their guidelines and proposed to expand hepatectomy is feasible for HCC patients with portal hypertension or elevated bilirubin but controllable ascites or treatable esophageal varices (Fig. 1). Nonetheless, the surgical issue refers to the comprehensive integration CSPH grades, liver function and extend of hepatectomy into the surgical principal remains uncertain. This study was therefore designed to shed light this matter and to provide a basis for rational and precise surgical decisions for individual HCC patients with CSPH.

## Methods

### Patients

Patients who underwent liver resection as initial treatment for HCC were considered for this retrospective study. The inclusion criteria were as follows: (1) underwent curative hepatectomy for HCC; (2) preoperative Eastern Cooperative Oncology Group (ECOG) performance score 0–1 and Child-Pugh score$\le$7; (3) post-operative histology examination confirmed of HCC; (4) did not receive any preoperative anticancer treatments; (5) without cardiopulmonary, renal, or cerebral dysfunction; (6) without other malignant tumors; (7) without incomplete clinical information.

According to the above criteria, a total of 555 HCC patients between April 2017 and June 2020 at the Guangxi Medical University Cancer Hospital (GXMUCH), and 369 HCC patients between September 2012 and June 2019 at the Eastern Hepatobiliary Surgery Hospital (EHBH) were included. This study was approved by the Institutional Ethics Committees of the two hospitals and was conducted in accordance with the principles stated in the Helsinki Declaration. Furthermore, written informed consents were acquired from all participating patients.

### Definitions

Chronic hepatitis B virus (HBV) infection was defined as hepatitis B surface antigen positive and can be detected with elevated HBV-DNA levels for more than 6 months [20]. In this study, patients with chronic HBV infection were advised to receive antiviral therapy. The definition and grading of PHLF referred to International Study Group of Liver Surgery (ISGLS) criteria [21]. Patients with an increased international normalized ratio (INR) and increased serum total bilirubin on or after postoperative day 5 is considered as suffered PHLF. Grade A PHLF did not require any treatment; grade B PHLF required noninvasive treatments, such as fresh frozen plasma and albumin transfusions; grade C PHLF required invasive treatments, such as mechanical ventilation, hemodialysis, and extracorporeal liver support. In this study, grade B and grade C PHLF were defined as severe PHLF [22, 23]. CSPH was defined as the presence of gastric and/or esophageal varices detectable by endoscopy and/or computed tomography, and the presence of splenomegaly (pedicle rib unit > 5) with a platelet (PLT) count of < 100$\times$10^9^/L [1]. Mild CSPH was defined as the presence of gastric and/or esophageal varices alone, or splenomegaly plus PLT count < 100$\times$10^9^/L alone; and severe CSPH as with gastric and/or esophageal varices combined with splenomegaly plus PLT count < 100$\times$10^9^/L [5]. Postoperative mortality was defined as death within 90 days after surgery [22].

### Preoperative examinations and surgical procedure

Preoperative serum examinations (including liver and renal functions, hepatitis immunology, serum α-fetoprotein level), abdominal contrast-enhance computed tomography or magnetic resonance imaging scan were routinely conducted. Indocyanine green retention rate at 15 min (ICG-R15) and standard future liver remnant (sFLR) was routinely calculated before surgery. The sFLR was calculated as [24]: FLR/estimated total liver volume (eTLV). The eTLV (cm^3^) was calculated as [25]: 706.2×body surface area (BSA) + 2.4. The BSA (m^2^) was calculated as [26]: 0.0126×weight (kg) + 0.00586× height (cm)-0.0461 for women and 0.0127× weight (kg) + 0.00607×height (cm)-0.0698 for men. The details of ICG-R15, sFLR and surgical procedures have been in our previous study [23, 27]. The liver, kidney, and coagulation function tests were conducted at 1, 3, 5, and 7 days after hepatectomy, or more frequently as appropriate.

### Study design and statistical analyses

The flow chart of the study design was shown in Supplemental Fig. 1. A stratified random grouping method was performed to randomly divided patients from our centre into a training cohort and a internal validation cohort at a ratio of 7:3. The significance of each variable in the training cohort was assessed by univariate logistic regression analysis to identify the risk factors of severe PHLF which were then classified according to clinical significance in seven groups. Stochastic according to the forest algorithm, indexes with the highest weight in each category were extracted and incorporated into the subsequent multivariate logistic regression. A nomogram was then established based on the results from multivariate logistic regression. The calibration capacity of the nomogram was tested via calibration plot.The predictive capacity of the nomogram was assessed using the area under receiver operating characteristic curve (AUC) and compared with common scores. The clinical benefit of the nomogram was calculated by decision curve analysis (DCA). Considering the PHLF can indeed be misjudged, patients with total points fall within the 95% CI misdiagnosis threshold were extracted for further determination by a conditional inference tree (CTREE). These results were also validated in an external validation cohort.

Data statistical analyses were performed using SPSS (v25.0) and R software (v4.0.2). Non-normal distributed data were represented as median (IQR 25–75) and compared using Mann-Whitney U test. Normally distributed data were expressed as mean ± standard deviation and compared using t tests. Categorical data were shown as frequency(proportion) and compared using χ^2^ test. All statistical tests were two-tailed, and *P* values < 0.05 were considered statistically significant.

## Results

### Clinicopathological characteristics

**Fig. 2:**
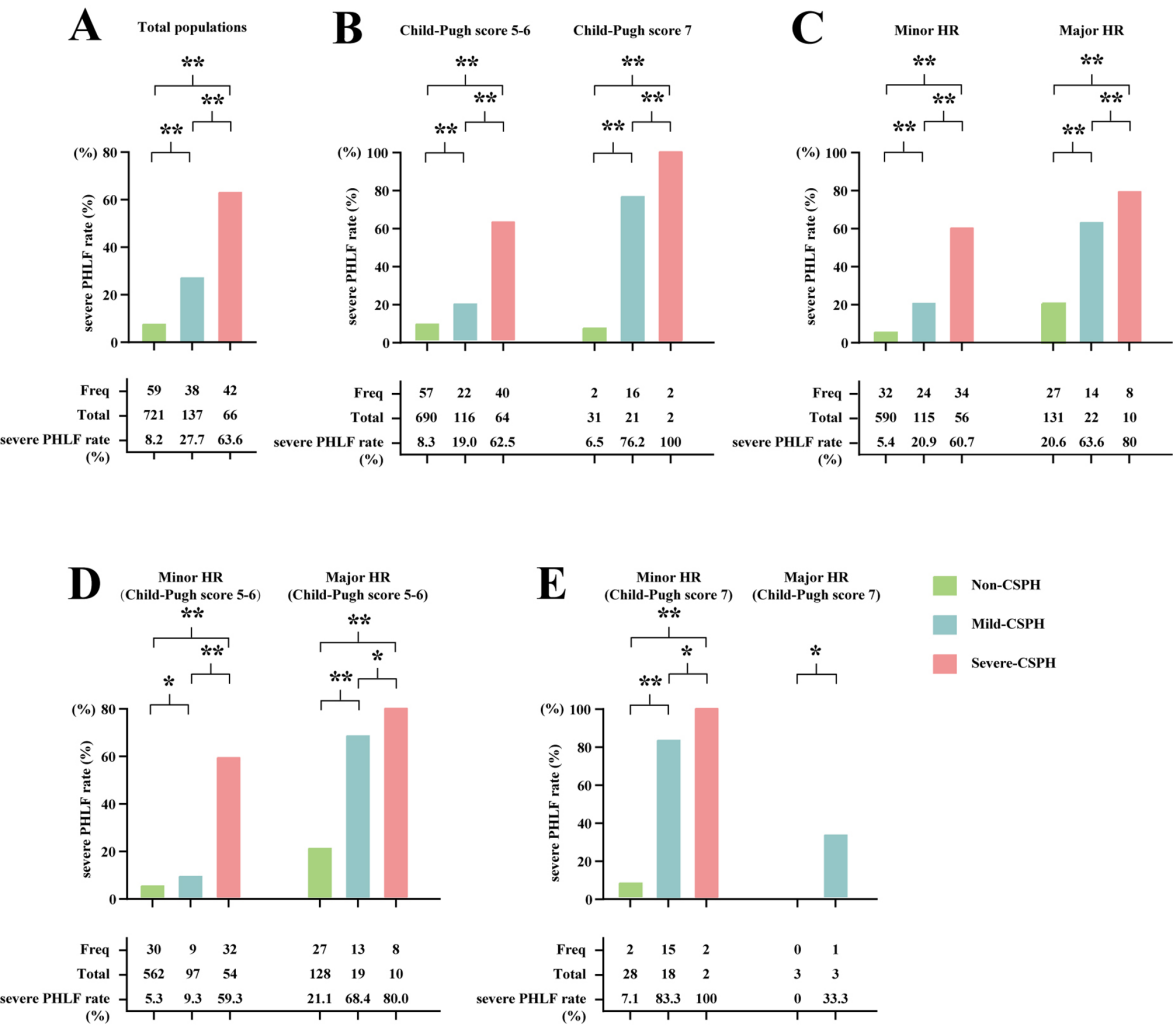
The incidence of severe PHLF were compared among non-CSPH, mild-CSPH and severe-CSPH groups; **(A)** total populations; **(B)** Child-Pugh score 5–6 or Child-Pugh score 7 patients; **(C)** patients who underwent minor or major HR; **(D)** Child-Pugh score 5–6 patients who underwent minor or major HR; **(E)** Child-Pugh score 7 patients who underwent minor or major HR. ∗>0.05, ∗∗< 0.05. *Abbreviations*: PHLF, post-hepatectomy liver failure; CSPH, clinically significant portal hypertension; HR, hepatic resection

The clinicopathological characteristics of included patients in the training cohort, internal validation cohort, and external validation cohort are summarized in Table 1. The major etiology of HCC was HBV infection, accounting for 80.6% of the entire study population. According to the CSPH gradation criteria, 78.0% of the patients (721/924) were assigned to non-CSPH group, 14.9% (137/924) to mild-CSPH group, and 7.1% (66/924) to severe-CSPH group. The comparison of incidence of severe PHLF among patients in terms of without CSPH, mild-CSPH and severe-CSPH were showed in Fig. 2.

**Table 1:** Demographics and clinicopathologic characteristics of study participants

Variables	Entire patients (*n* = 924)	Training Cohort (*n* = 389)	Internal validation Cohort (*n* = 166)	External validation Cohort (*n* = 369)
Age (years)	52 ± 11	52 ± 11	51 ± 11	53 ± 10
Male, n (%)	816 (88.3%)	347 (89.2%)	144 (86.7%)	325 (88.1)
Height (cm)	165 (161, 170)	165 (160, 170)	166 (162, 170)	165 (161, 170)
Weight (kg)	61 (54, 68)	60.5 (55.0, 68.0)	61 (54, 68)	60 (54,69)
Positive HBsAg, n (%)	745 (80.6%)	312 (80.2%)	135 (81.3%)	298 (80.8%)
PLT (× 10^9^/L)	178.5 (134.3, 219.8)	194.0 (148.0, 246.0)	185.5 (144.0, 249.3)	148.0 (96.0, 192.5)
T-Bil (µmol/L)	15.6 (11.5, 20.1)	16.1 (11.5, 20.7)	15.8 (12.2, 19.6)	15.3 (11.2, 20.6)
PA (mg/L)	180.0 (136.0, 222.0)	171.0 (131.5, 211.0)	170.5 (133.5, 207.5)	190.0 (140.5, 236.0)
ALB (g/L)	38.8 ± 5.4	37.5 ± 4.8	38.6 ± 6.9	41.1 ± 4.5
ALT (U/L)	37.0 (27.0, 59.0)	39.0 (28.0, 59.0)	39.0 (26.0, 61.0)	34.0 (25.0, 57.0)
AST (U/L)	41.0 (29.0, 65.0)	44.0 (31.0, 68.0)	49.0 (33.0, 75.0)	35.0 (26.0, 54.9)
CR (µmol/L)	69.0 (61.0, 78.0)	69.0 (60.0, 78.5)	69.0 (61.0, 78.0)	68.0 (60.0, 78.0)
PT (s)	12.5 (11.7, 13.3)	12.5 (11.7, 13.3)	13.0 (12.4, 13.8)	12.1 (11.5, 12.9)
INR	1.03 (0.97, 1.10)	1.03 (0.96, 1.09)	1.09 (1.03, 1.16)	1.01 (0.97, 1.08)
AFP (ng/mL), n (%)				
≥ 400	352 (38.1%)	156 (40.1%)	71 (42.8%)	125 (33.9%)
< 400	572 (61.9%)	233 (59.9%)	95 (57.2%)	244 (66.1%)
CSPH grade				
No	721 (78.0%)	326 (83.8%)	132 (79.5%)	263 (71.3%)
Mild	137 (14.9%)	43 (11.1%)	31 (18.7%)	63 (17.1%)
Severe	66 (7.1%)	20 (5.1%)	3 (1.8%)	43 (11.7%)
ICG-R15 (%)	4.9 (3.2, 8.0)	4.8 (3.3, 7.8)	5.0 (3.2, 7.9)	5.1 (3.1, 8.3)
Child-Pugh grade, n (%)				
A	870 (94.2%)	365 (93.8%)	151 (91.0%)	354 (95.9%)
B	54(5.8%)	24 (6.2%)	15 (9.0%)	15 (4.1%)
MELD score	4.0 (2.1, 6.0)	4.0 (2.1, 6.0)	4.6 (2.7, 6.2)	3.8 (2.0, 5.9)
ALBI score	-2.54 ± 0.48	-2.38 ± 0.41	-2.50 ± 0.58	-2.71 ± 0.41
PALBI score	-2.41 (-2.64, -2.18)	-2.31 (-2.50, -2.10)	-2.36 (-2.53, -2.16)	-2.59 (-2.78, -2.35)
APRI score	0.62 (0.40, 1.06)	0.59 (0.39, 0.92)	0.65 (0.45, 1.10)	0.69 (0.38, 1.20)
FIB-4	2.07 (1.37, 3.31)	1.99 (1.35, 2.97)	2.11 (1.43, 3.54)	2.18 (1.37, 3.81)
Tumour size (cm)	6.5 (4.0, 10.0)	7.0 (4.5, 10.0)	7.0 (4.1, 12.0)	6.0 (4.0, 10.0)
Tumour number, n (%)				
Multiple	109 (11.8%)	55 (14.1%)	29 (17.5%)	25 (6.8%)
Single	815 (88.2%)	334 (85.9%)	137 (82.5%)	344 (93.2%)
Portal invasion, n (%)	92 (10.0%)	45 (11.6%)	17 (10.2%)	30 (8.1%)
Operation time (min)	205 (170, 250)	210 (180, 250)	220 (172, 270)	190 (160, 230)
Blood loss (mL), n (%)				
≥ 400	327 (35.4%)	117 (30.1%)	71 (42.8%)	139 (37.7%)
< 400	597 (64.6%)	272 (69.9%)	95 (57.2%)	230 (62.3%)
Blood transfusion, n (%)	130 (14.4%)	61 (15.7%)	25 (15.1%)	47 (12.7%)
Extent of resection, n (%)				
Major	163 (17.6%)	91 (23.3%)	57 (34.3%)	15 (4.1%)
Minor	761 (82.4%)	298 (76.7%)	109 (65.7%)	354 (95.9%)
sFLR (%)	68.8 (55.7, 80.0)	64.4 (51.2, 77.2)	60.0 (45.9, 77.0)	77.0 (67.0, 85.0)
Hepatic vascular occlusion				
No	227 (24.6%)	92 (23.7%)	36 (21.7%)	99 (26.8%)
HVC	275 (29.8%)	117 (30.1%)	48 (28.9%)	110 (29.8%)
THVE	422 (45.7%)	180 (46.3%)	82 (49.4%)	160 (43.4%)
Cirrhosis, n (%)	468 (50.6%)	204 (52.4%)	87 (52.4%)	177 (48.0%)
PHLF Grade, n (%)	273 (29.5%)	111 (28.5%)	46 (27.7%)	116 (31.5%)
A	134 (14.5%)	48 (12.3%)	26 (15.7%)	60 (16.3%)
B	128 (13.9%)	58 (14.9%)	19 (11.4%)	51 (13.8%)
C	11 (1.1%)	5 (1.3%)	1 (0.6%)	5 (1.4%)
Severe PHLF, n (%)	139 (15.0%)	63 (16.2%)	20 (12.0%)	56 (15.2%)
90-d mortality, n (%)	11 (1.2%)	4 (1.0%)	2 (1.2%)	5 (1.4%)

Note: Data are mean ± SD or median (IQR 25–75) unless otherwise indicated

Abbreviations: HbsAg, hepatitis B surface antigen; PLT, platelet; T-Bil, total bilirubin; PA, prealbumin; ALB, albumin; ALT, alanine aminotransferase; AST, aspartate aminotransferase; CR, creatinine; PT, prothrombin time; INR, international normalized ratio; ICG-R15, indocyanine green retention rate at 15 min; MELD, model for end-stage liver disease; ALBI, albumin–bilirubin; PALBI, platelet-albumin-bilirubin; APRI, aspartate aminotransferase to platelet ratio index; AFP, α-Fetoprotein; CSPH, clinically signifcant portal hypertension; BCLC, Barcelona Clinic Liver Cancer; sFLR, standard future Liver remnant; HVC, hemilhepatic vascular control; THVE, total hepatic vascular exclusion; PHLF, post-hepatectomy liver failure

### Postoperative morbidity and mortality

A total of 139 patients (15.0%) developed severe PHLF in this study, including 63 patients (16.2%) in the training cohort, 20 patients (12.0%) in the internal validation cohort, and 56 patients (15.2%) in the external validation cohort(Table 1). The overall incidence of 90-day mortality was 1.2% in this study, including 1.6% in the training cohort, 2.0% in the internal validation cohort, and 1.4% in the external validation cohort (Table 1). Among the entire patients, 90-days mortality in patients with severe PHLF was greatly higher than that in patients without severe PHLF (5.3% vs. 0.5%; *P* < 0.001), and 90-days mortality was also significantly higher in patients with severe CSPH than in patients with mild CSPH and without CSPH (6.1% vs. 2.2% vs. 0.6%, *P* < 0.001).

### Development and validation of a nomogram for determining candidates for hepatectomy

**Fig. 3:**
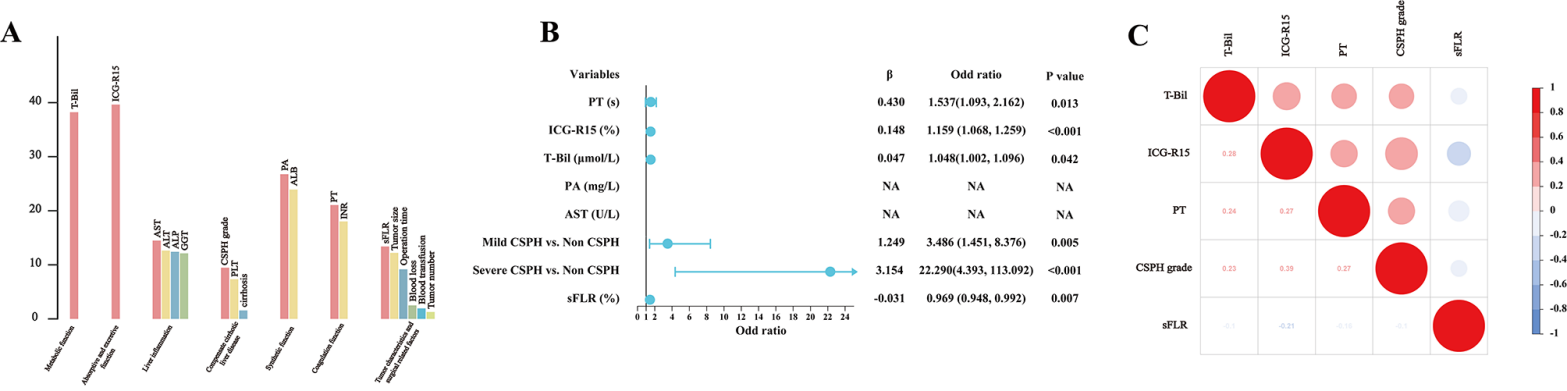
**(A)** The importance of the Stochastic Forest algorithm based on grouping indexes. Logistic univariate significant indicators were divided into seven groups according to clinical significance and a random forest model was constructed for each group of indicators to predict severe PHLF risk. The bars represent the importance of each indicator; the red bars represent the most important indicators of each group. **(B)** Multivariate logistic regression analyses to identify predictors of severe PHLF in HCC patients in the training cohort. Forest maps show the risk ratios of indicators. **(C)** There is no correlation among the indicators after redundancy removal by grouping stochastic forest algorithm. Colors from red to blue indicate a correlation from positive to negative. The values inside the circle represent the significant *P* values of the correlations, indicating the correlations among all indicators are not significant. *Abbreviations*: PHLF, post-hepatectomy liver failure; CSPH, clinically significant portal hypertension; HR, hepatic resection; HCC, hepatocellular carcinoma

**Fig. 4:**
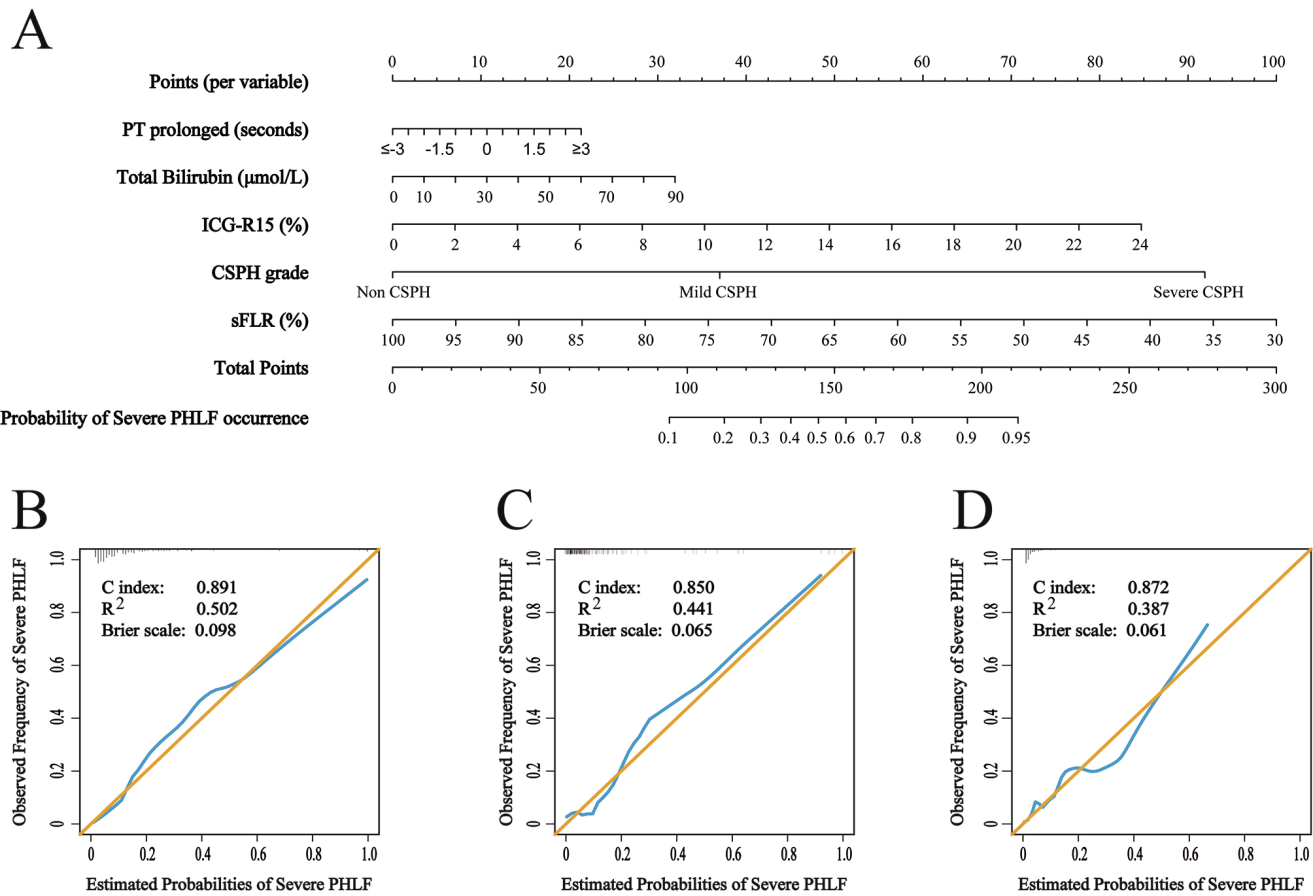
**(A)** The nomogram to predict severe PHLF was created based on 5 independent prognostic factors. **(B)** Validity of the predictive performance of the nomogram in estimating the risk of severe PHLF in the training cohort (*n* = 389). **(C)** Validity of the predictive performance of the nomogram in estimating the risk of severe PHLF in the internal validation cohort (*n* = 166). **(D)** Validity of the predictive performance of the nomogram in estimating the risk of severe PHLF in the external validation cohort (*n* = 369). *Abbreviations*: PHLF, post-hepatectomy liver failure

In the training cohort, risk factors associated with severe PHLF were identified by univariate logistic analysis (Table 2). And the data redundancy and excessive false positives were eliminated by correlation analysis and forest algorithm (Fig. 3A). Multivariate logistic analysis with stepwise removal of variables was then conducted. The results were reported as OR with 95%CI, and revealed that prolonged prolonged prothrombin time (PT), ICG-R15, T-Bil, CSPH grade, sFLR were independently corresponded to severe PHLF and correlation test revealed no significant interdependence among them (Fig. 3B and C). Then, a nomogram integrating these factors was generated (Fig. 4A). The calibration curve showed a good agreement between the likehood of severe PHLF using the nomogram and its actual observed incidence of the disease in the training cohort (*R*^2^ = 0.502), internal validation cohort (R^2^ = 0.441), and external validation cohort (*R*^2^ = 0.387) (Fig. 4B-D). The C-index for prediction of severe PHLF was 0.891 (95%CI: 0.855–0.920) for the training cohort, 0.850 (95%CI: 0.786–0.901) for the internal validation cohort, and 0.872 (95%CI: 0.835–0.904) for the external validation cohort (Fig. 4B-D). In the training cohort, the optimal cutoff value of total point to predict severe PHLF was determined to be 110.5, with a sensitivity, specificity, positive predictive value, and negative predictive value of 83.9%, 82.8%, 56.2%, and 95.1%, respectively. Bootstrap validation results showed good performance, with a sensitivity, specificity, positive predictive value, and negative predictive value of 80.0%, 71.2%, 27.6%, and 96.3% in the internal validation cohort; and 78.4%, 82.9%, 32.6% and 97.3% in the external validation cohort (Table 3).

**Table 2:** Univariable logistic analyses to identify predictors for severe PHLF in the training cohort

Variables	β^a^	Odds Ratio (95%CI)	*P* value
Age, years	0.004	1.004 (0.980, 1.028)	0.773
Male	-0.550	0.577 (0.268, 1.243)	0.160
Weight, kg	-0.027	0.973 (0.934, 1.014)	0.190
Height, cm	-0.009	0.991 (0.964, 1.018)	0.504
Positive HBsAg	0.116	1.123 (0.539, 2.339)	0.757
ICG-R15, %	0.250	1.283 (1.200, 1.373)	< 0.001
AFP, ≥ 400ng/mL	0.058	0.836 (0.612, 1.833)	0.836
PLT count, 10^9^/L	-0.005	0.995 (0.991, 0.998)	0.016
PT, s	0.764	2.146 (1.643, 2.803)	< 0.001
INR, $\ge$1.2	1.240	3.456 (1.440, 8.293)	0.006
ALB, g/L	-0.086	0.917 (0.856, 0.983)	0.015
PA, mg/L	-0.010	0.990 (0.985, 0.995)	< 0.001
ALT, U/L	0.009	1.010 (1.002, 1.017)	0.014
AST, U/L	0.013	1.013 (1.005, 1.022)	0.001
ALP, U/L	0.003	1.003 (1.001, 1.006)	0.046
GGT, U/L	0.002	1.002 (1.001, 1.005)	0.048
CR, µmol/L	-0.005	0.995 (0.977, 1.015)	0.638
T-Bil, µmol/L	0.097	1.102 (1.062, 1.143)	< 0.001
Child-Pugh grade, B	-0.320	0.726 (0.210, 2.512)	0.613
Cirrhosis	1.249	3.487 (1.878, 6.477)	< 0.001
CSPH grade			
Without CSPH	Reference	Reference	NA
Mild CSPH	1.665	5.286 (2.545, 10.979)	< 0.001
Severe CSPH	4.486	88.800 (19.649, 401.318)	< 0.001
Tumor size, cm	0.075	1.078 (1.002, 1.160)	0.043
Tumor number, multiple	0.804	2.234 (1.146, 4.356)	0.018
Portal invasion	-0.752	0.471 (0.163, 1.366)	0.166
sFLR, %	-0.049	0.952 (0.934, 0.971)	< 0.001
Operation time (min)	0.001	1.001 (0.997, 1.005)	0.605
Blood loss, ≥ 400mL	0.676	1.967 (1.129, 3.426)	0.017
Blood transfusion	0.754	2.126 (1.111, 4.067)	0.023
Hepatic vascular occlusion			
No	Reference	Reference	NA
HVC	-0.894	0.409 (0.164, 1.022)	0.056
THVE	0.497	1.643 (0.843, 3.202)	0.144

Abbreviations: HbsAg, hepatitis B surface antigen; PLT, platelet; T-Bil, total bilirubin; PA, prealbumin; ALB, albumin; ALT, alanine aminotransferase; AST, aspartate aminotransferase; CR, creatinine; PT, prothrombin time; INR, international normalized ratio; ICG-R15, indocyanine green retention rate at 15 min; AFP, α-Fetoprotein; CSPH, clinically signifcant portal hypertension; BCLC, Barcelona Clinic Liver Cancer; sFLR, standard future Liver remnant; HVC, hemilhepatic vascular control; THVE, total hepatic vascular exclusion; PHLF, post-hepatectomy liver failure; NA, not available

**Table 3:** Accuracy of the nomogram for estimating the risk of severe PHLF

Variable	Value (95% CI)		
	Training cohort	Internal validation cohort	External validation cohort
Area under ROC curve	0.891 (0.855 to 0.920)	0.850 (0.786 to 0.901)	0.872 (0.835 to 0.904)
Cutoff score	110.5	110.5	110.5
Sensitivity, %	83.9 (74.1 to 91.2)	80.0 (56.3 to 94.3)	78.4 (61.8 to 90.2)
Specificity, %	82.8 (78.1 to 86.8)	71.2 (63.2 to 78.4)	82.9 (78.5 to 86.7)
Positive predictive value, %	56.2 (49.7 to 62.5)	27.6 (21.4 to 34.8)	32.6 (26.6 to 39.1)
Negative predictive value, %	95.1(92.2 to 97.0)	96.3 (91.5 to 98.4)	97.3 (95.1 to 98.5)
Positive likelihood ratio	4.9 (3.8 to 6.3)	2.8 (2.0 to 3.9)	4.6 (3.4 to 6.1)
Negative likelihood ratio	0.19 (0.1 to 0.3)	0.28 (0.1 to 0.7)	0.26 (0.1 to 0.5)

**Fig. 5:**
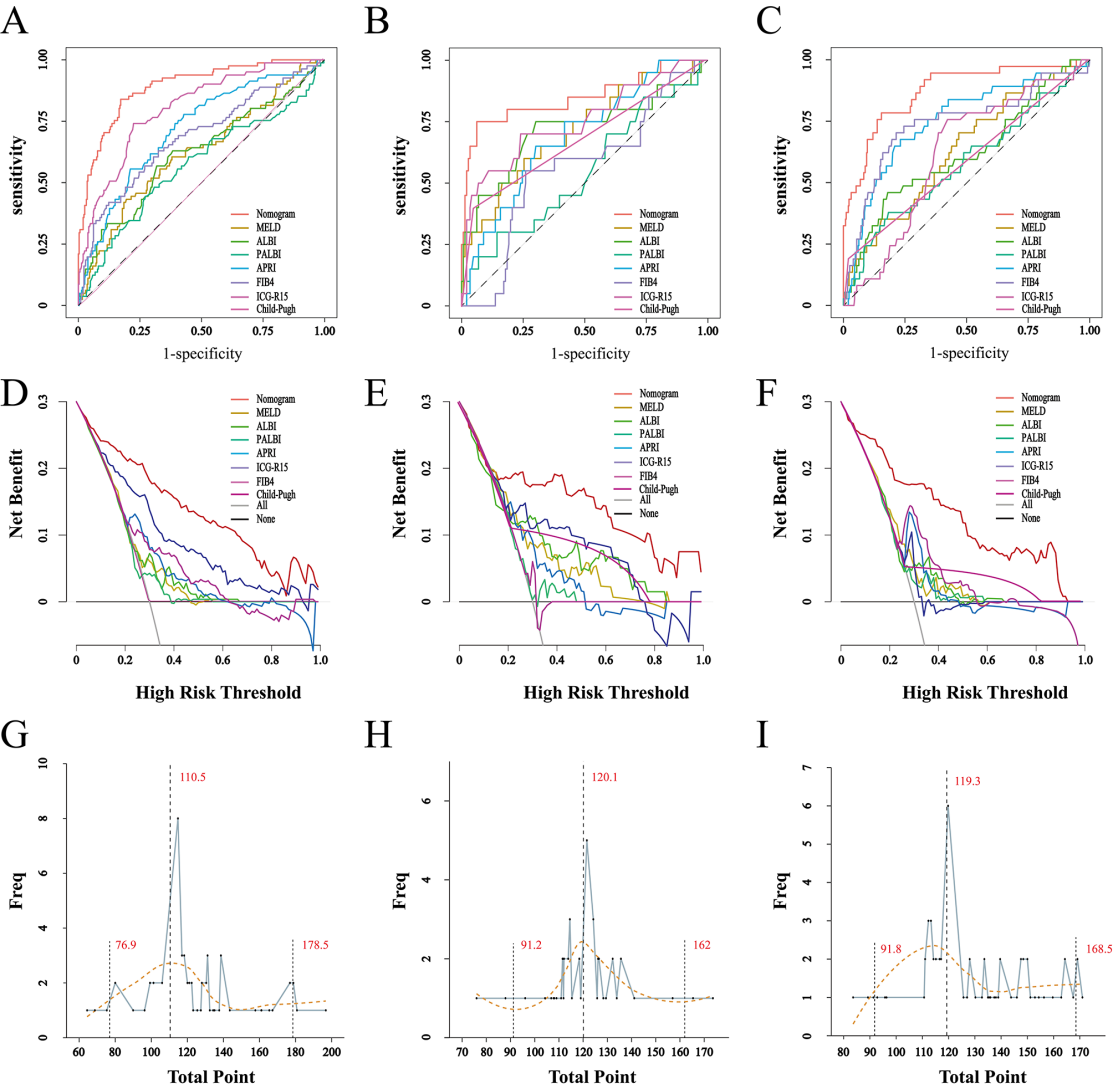
ROC curves for the nomogram and other commonly used scoring systems to predict the risk of severe PHLF in the **(A)** training cohort, **(B)** internal validation cohort, **(C)** external validation cohort. Decision curve analysis of the nomogram and other conventional scores in the **(E)** training cohort, **(F)** internal validation cohort, **(G)** external validation cohort. The x-axis represents the threshold probability. The y-axis represents the net benefit. **(G-I)** Total points distribution of false positive events (blue polyline). The X-axis represents the total points used to predict the risk of severe PHLF, and the Y-axis represents the frequency of false positive events. The yellow dotted line represents the fitted line and presents normal distribution. **(G)** Training cohort, the false positive events were concentrated around the maximum value of 110.5 point. **(H)** Internal validation cohort, the false positive events were concentrated around the maximum value of 120.1 point. **(I)** External validation cohort, the false positive events were concentrated around the maximum value of 119.3 point. *Abbreviations*: PHLF, post-hepatectomy liver failure; ROC, receiver operating characteristic

This nomogram showed a superior determining performance of severe PHLF that the AUCs under the nomogram were greater than MELD, ALBI, PALBI, APRI, FIB-4, ICG-R15 and Child-Pugh class among the training, internal validation and external validation cohort (Tables 4**and** Fig. 5A-C). The DCA was used to facilitate the comparison of clinical usefulness between the nomogram and other conventional scores. The nomogram showed a superior net benefit across a wider scale of threshold probabilities for predicting severe PHLF than the conventional scores (Fig. 5D-F). Calculations for the objectivity evaluation of the diagnostic CI revealed that total points of diagnostic errors with 95%CI were concentrated in 110.5 (ranged 76.9-178.5) in the training cohort; 120.1 (ranged 91.2–162) in the internal validation cohort; and 119.3 (ranged 91.8-168.5) in the external validation cohort (Fig. 5G-I) that the concentrated total points were all concentrated close to the best cutoff value of 110.5 among the three cohorts. It supposed a low risk of severe PHLF and hepatectomy is feasible when total points fall below this range, while it supposed a very high risk of severe PHLF and hepatectomy is absolutely restricted when total points are beyond than this range. However, when total points fall within this range, the prediction results should be carefully considered.

**Table 4:** Comparison of different models in predicting severe PHLF

	Training cohort				Internal validation cohort				External validation cohort		
Models	AUC	95% CI	*P* value		AUC	95% CI	*P* value		AUC	95% CI	*P* value
Nomogram model	0.891	0.855–0.920	< 0.001		0.850	0.786–0.901	< 0.001		0.872	0.831–0.906	< 0.001
MELD score	0.621	0.550–0.671	0.001		0.715	0.589–0.841	0.002		0.34	0.542–0.727	0.007
ALBI score	0.618	0.544–0.692	0.001		0.717	0.517–0.864	0.002		0.617	0.514–0.720	0.019
PALBI score	0.570	0.494–0.646	0.053		0.547	0.405–0.688	0.498		0.577	0.472–0.682	0.124
APRI score	0.715	0.651–0.778	< 0.001		0.692	0.573–0.812	0.005		0.750	0.662–0.838	< 0.001
FIB-4 score	0.684	0.614–0.753	< 0.001		0.563	0.430–0.696	0.361		0.739	0.640–0.838	< 0.001
Child-Pugh grade	0.497	0.426–0.567	0.926		0.576	0.440–0.712	0.243		0.586	0.479–0.693	0.085
ICG-R15	0.802	0.749–0.855	< 0.001		0.751	0.621–0.881	< 0.001		0.616	0.530–0.703	0.020

Abbreviation: AUC, area under the ROC curve; CI, confidence interval; MELD, model for end-stage liver disease; ALBI, albumin-bilirubin; PALBI, platelet-albumin-bilirubin; FIB-4, fibrosis index based on the 4 factors; APRI, aspartate aminotransferase to platelet ratio index; PHLF, post-hepatectomy liver failure

### Development of conditional inference tree

Considering the severe-PHLF risk can indeed be misjudged, patients whose total points fall within the misdiagnosis threshold range were extracted and further analyzed by a CTREE (Supplemental Fig. 2). The importance of each predictor variables of severe PHLF including PT, T-Bil, ICG-R15, CSPH grade, and sFLR were ranked based on the conditional variable importance analysis. Of all the variables examined, CSPH grade was identified as the best discriminator, and the subsequent splits of severe PHLF were ICG-R15 and sFLR which would stratifiy participants into 19 nodes, PT and T-Bil were excluded as irrelevant in the CTREE.

**Fig. 6:**
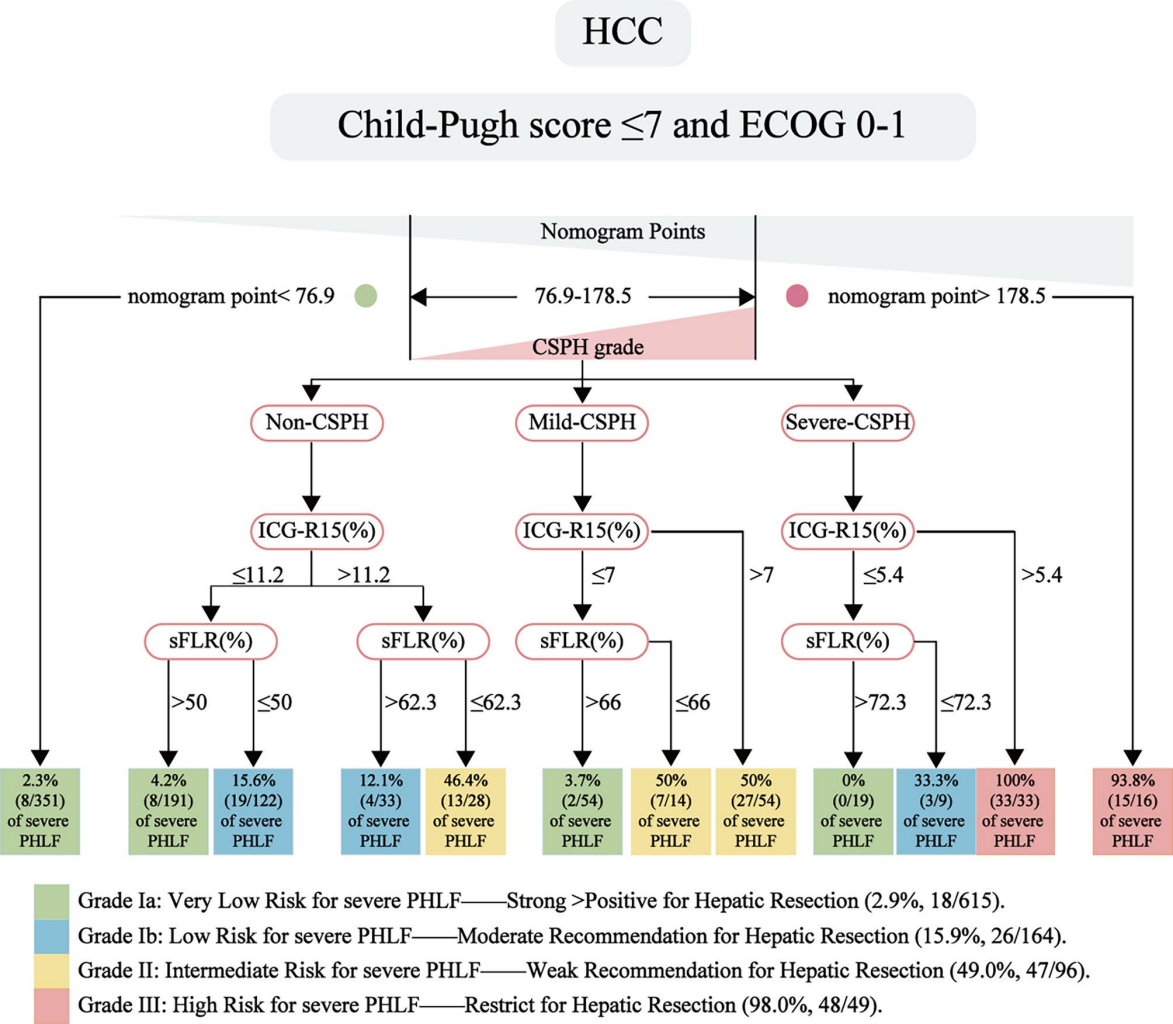
A new surgical scheme includes a range from very low-risk to high-risk subsets through integrating nomogram and CTREE model for HCC patients with or without CSPH. *Abbreviations*: CSPH, clinically significant portal hypertension; HCC, hepatocellular carcinoma; CTREE, conditional inference tree

These branch points stratified participants into 19 nodes according to their exact severe-PHLF risk as follows (Fig. 6): (i) severe-CSPH and ICG-R15$\le$5.4%, followed by sFLR$\le$72.3% (33.9% suffered severe PHLF) and sFLR>72.3% (0% suffered severe PHLF). (ii) severe-CSPH followed by ICG-R15>5.4% (100% suffered severe PHLF). (iii) mild-CSPH and ICG-R15$\le$7%, followed by sFLR$\le$66% (50% suffered severe PHLF) and sFLR>66% (3.7% suffered severe PHLF). (iv) mild-CSPH followed by ICG-R15>7% (50% suffered severe PHLF). (v) non-CSPH and ICG-R15$\le$11.2%, followed by sFLR$\le$50% (15.6% suffered severe PHLF) and sFLR>50% (4.2% suffered severe PHLF). (vi) non-CSPH and ICG-R15>11.2%, followed by sFLR$\le$62.3% (46.4% suffered severe PHLF) and sFLR > 62.3% (12.1% suffered severe PHLF). The CTREE offer a straightforward visualization to assign participants into different groups ranged from very low- to high-risk, supplemented the uncertain diagnostic threshold probabilities of the nomogram for the surgical decision-making procedure.

## Discussion

Surgical decisions on identifying favorable candidates for hepatectomy involve maintaining a balance between the expected long-term oncological outcomes of HCC removal on one hand, and the short-term risk of PHLF and morbidity on the other. In the past few decades, although the BCLC (since 2018) [16], EASL (since 2012) [14], AASLD (since 2018) [15], NCCN (since 2009) [13], CNLC (since 2011) [3], JSH (since 2021) [19], and TLCA (since 2016) [18] had accepted hepatectomy to HCC, but merely expand minor hepatectomy to HCC patients with moderate CSPH and well-preserved liver function [10–12]. To date, individual hepatectomy for favorable candidates with specific CSPH grade and compensated liver function remains uncertain. Thus, a precise quantification of the risk for PHLF is imperative in surgical practice.

Measurement of hepatic venous pressure gradient (HVPG) via catheterization of the hepatic vein is the most reliable diagnostic criterion for CSPH [28], but this invasive procedure is not universal applied that prompted attempts to look for non-invasive alternatives [29]. In 1999, BCLC proposed a surrogate indirect criterion for CSPH (mentioned above) instead [6]. A prospective study [5] highlighted the significance of CSPH stratification. In that work, a total of 190 HCC patients were enrolled and stratified according to the surrogate indirect criteria of CSPH, patients with severe-CSPH possessed higher PVP, corresponding to higher risk of severe PHLF than those with non-CSPH or mild-CSPH. In this multi-center study, we adopted the surrogate indirect criteria of CSPH and found similar results that CSPH increased the risk of severe PHLF, particularly in the severe-CSPH group, confirming the reliability of severity of CSPH being associated with the post-operative outcomes. Also, we found no obvious difference between non-CSPH and mild-CSPH subgroups with respect to the incidence of severe PHLF in Child-Pugh A patients who underwent minor hepatectomy. This may indicate that solely refer CSPH grade to hepatectomy is unreliable.

In this study, we successfully established a new surgical scheme to determine individual hepatectomy for HCC patients with CSPH. This scheme can provide an accurate risk map for the development of severe PHLF, thus providing a useful tool for surgical decision making in clinical practice. Obviously, the nomogram showed that low risk of severe PHLF (2.3%) and hepatectomy is feasible when the nomogram points fall below 76.9; on the contrary, the risk of severe PHLF is extremely high (93.8%) and hepatectomy should be rigorously restricted when the nomogram points are higher than 178.5. However, although the nomogram outperformed other systems in predicting severe PHLF, such as Child-Pugh, MELD, ALBI, PALBI, APRI, FIB-4, and ICG-R15, its diagnostic value may be misjudgment when the nomogram point falls within the misdiagnosis threshold range of 76.9 to 178.5. To assess the nomogram’s utility, the hierarchical interplay of prognostic factors for severe PHLF in patients falling within the nomogram’s misdiagnosis threshold range was further examined using CTREE. This analysis was conducted on a cohort of 557 HCC resections, categorizing patients into four subgroups that correlated with a significant increase in severe PHLF (2.9%, 15.9%, 49.0% and 98.0%, *p* < 0.05). For patients with absence of CSPH, when ICG-R15 ≤ 11.2%, sFLR > 50% vs.≤50% separated the likelihood of severe PHLF into a very low- vs. low-risk group; while ICG-R15 > 11.2%, sFLR > 62.3% vs.≤62.3% separated the likelihood of severe PHLF into a low- vs. intermediate-risk group. For patients with mild-CSPH, ICG-R15 > 7% directly indicated the severe PHLF into the intermediate-risk group or ICG-R15 ≤ 7% followed by sFLR > 66% vs.≤66% separated the likelihood of severe PHLF into a very low- vs. intermediate-risk group. In addition, for patients with severe-CSPH, ICG-R15 > 5.4% was considered as high risk of severe PHLF, while ICG-R15 ≤ 5.4% remained separated the likelihood of severe PHLF according to sFLR > 72.3% vs.≤72.3% into a very low- vs. low-risk group. The new surgical scheme provided a possible surgical strategy based on the risks of severe PHLF: highly recommending hepatectomy for suitable candidates with very low risk of severe PHLF; moderately suggesting hepatectomy for potential candidates with low risk of severe PHLF; cautiously proposing hepatectomy for eligible candidates with intermediate risk of severe PHLF; and strictly limiting hepatectomy in cases with a high risk of severe PHLF. Thus, such a relatively simple scheme built on nomogram and CTREE, as well as the risk stratification may offer an objective tool to simplify surgical decision making.

In 2016, the Liver Transplantation and Hepato-Biliopancreatic Surgery Unit of the National Cancer Institute of Milan proposed a prognostication tree recursive partitioning portal hypertension, followed by extension of hepatectomy and MELD score to identify tree risk classes closely associated with PHLF [30]. This model incorporated easy-to-access preoperative variables, which contributed to balanced decisions concerning liver resection for HCC, and it has been approved by the EASL [4]. This model did not indicate any relationship between CSPH grades and PHLF, nor did it integrate CSPH grades into their decisional algorithm. It used major/minor hepatectomy rather than explicit the sFLR, which has been widely accepted as the efficient prerequisite assessment to evaluate the functional remnant hepatic parenchyma [31]. However, the performance of MELD score at preoperative predicting PHLF remains controversial [32]. Instead, the ICG-R15 which is commonly used in the clinical evaluation of hepatic functional hepatocytes [1], did not take into account into their surgical scheme. Further, the model has not provided recommendations for individual hepatectomy in terms of the PHLF risk classes yet. In our study, we proposed a new scheme by generating a nomogram and CTREE to present the hierarchic interactions among very low-, low-, intermediate- and high-risk PHLF cases, contributing to the improvement of the predictive capacities. It appears capable of making strong, moderate, and weak and contraindications for individual hepatectomy in clinical applications.

Currently, hepatectomy is restricted to patients with the presence of severe-CSPH [1, 3, 4], which would potentially confine therapeutic improvement for HCC. However, with the application of our surgical scheme, the 19 patients with severe-CSPH were classified into very low-risk group and and 9 patients into low-risk group, for whom being the favorable/potential candidates for hepatectomy, demonstrating the surgical scheme would be practical to identify existing potential candidates with severe-CSPH to hepatectomy, thus directly expanding the indication of HCC to hepatectomy.

The current study remains several limitations. This new surgical scheme was derived based on the majority of population was associated with HBV infection, thus the predictive accuracy still needed to be further explored for other etiologies. In addition, the data sets included retrospective series, despite the unavoidable selection biases have been minimized by including a large cohort of consecutive patients, a large multi-center prospective study is also required to further confirm the reliability of this new surgical scheme.

## Conclusion

Our study establishing a new surgical scheme demonstrated the preoperative determination of severe PHLF risk can be stratified accurately by utilizing a nomogram and a CTREE according to a hierarchic order of factors represented by the presence of CSPH grades, extension of the hepatectomy, PT prolonged, T-Bil and the ICG-15 rates, and meanwhile provided the surgical strategy of individual hepatectomy for HCC patients with CSPH. This new surgical scheme potentially facilitated the surgical decision-making process could turn out to be significant to reduce or even eliminate postoperative mortality and improve the expected oncologic outcomes of HCC removal.

### Electronic supplementary material

Below is the link to the electronic supplementary material.

Supplementary Material 1. **Supplemental Fig. 1**. Flow chart of the study design.

Supplementary Material 2. **Supplemental Fig. 2**. The conditional inference tree of severe PHLF. The branch points stratified participants into 19 nodes according to risk of severe PHLF. *Abbreviations*: PHLF, post-hepatectomy liver failure.

## Data Availability

All data are available within the article and supplementary files, or available from the authors upon request.
