# Supplementary figures and images for: A novel surgical scheme for hepatectomy in hepatocellular carcinoma patients with clinically significant portal hypertension
Source: BMC Cancer. 2024 Jun 25;24:764. doi: 10.1186/s12885-024-12535-9 (PMC11202348; doi:10.1186/s12885-024-12535-9)

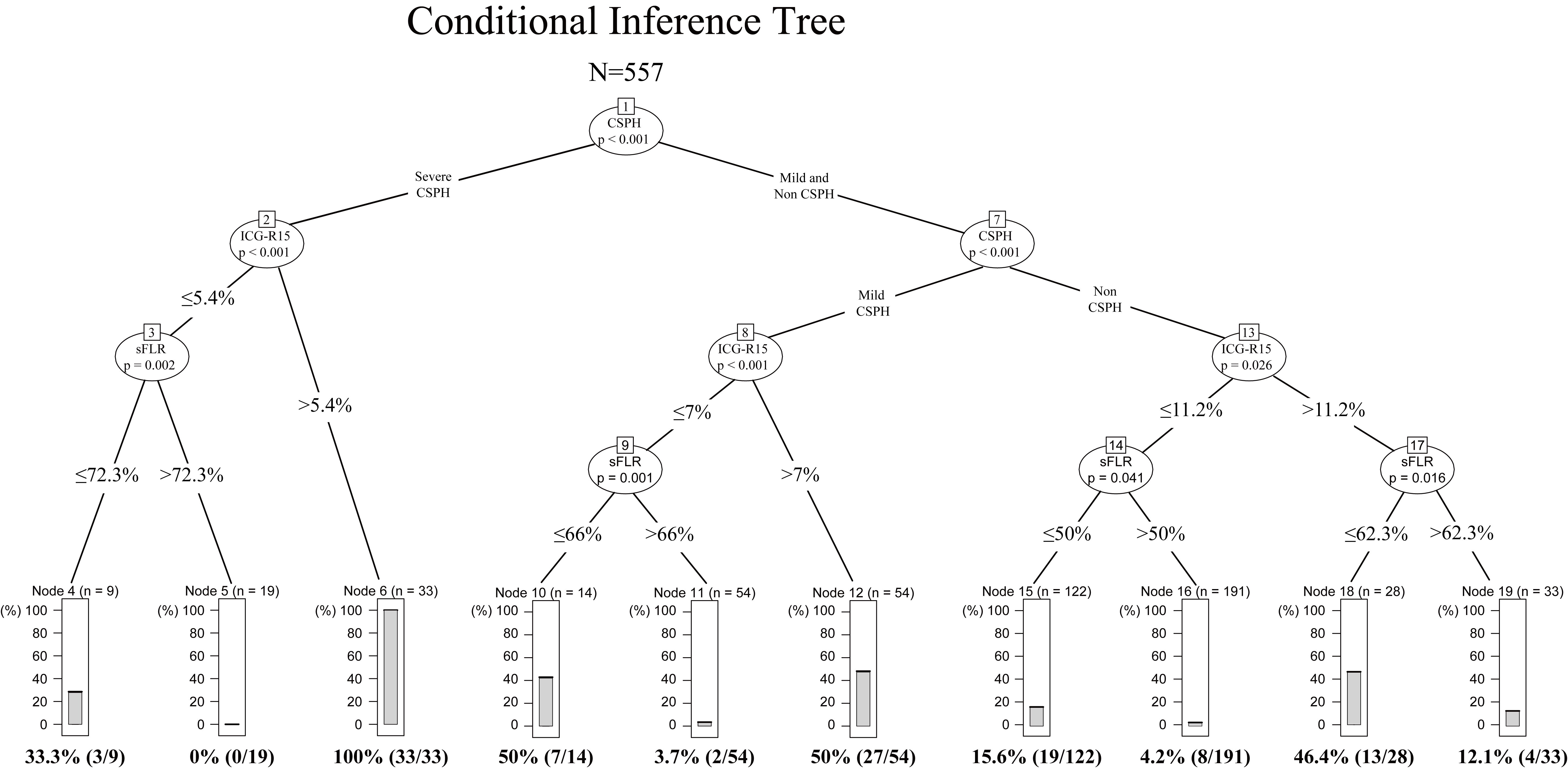

Supplement: Supplementary file 1 — Supplementary Material 1. Supplemental Fig. 1. Flow chart of the study design. [file 12885_2024_12535_MOESM1_ESM.jpg]

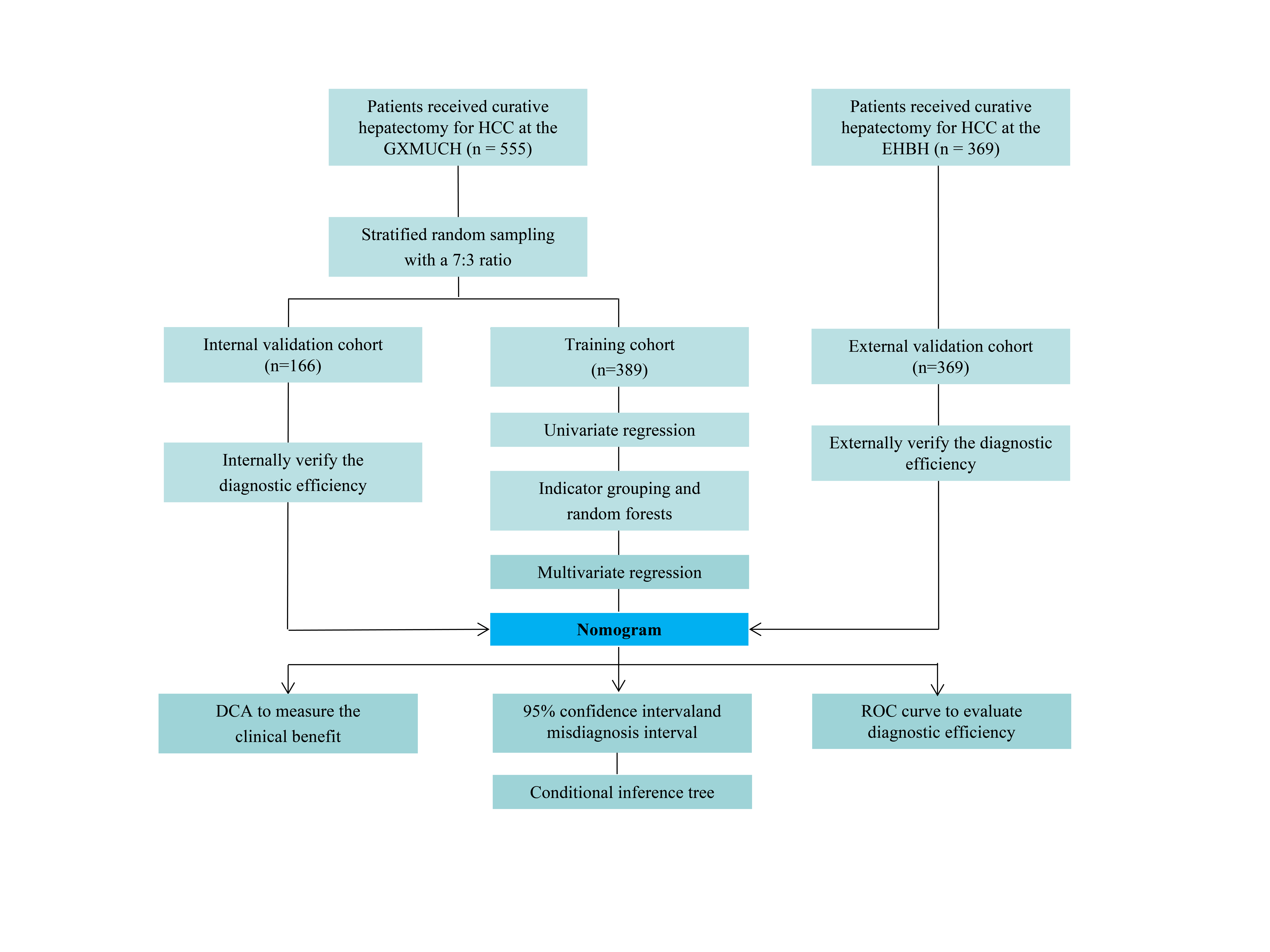

Supplement: Supplementary file 2 — Supplementary Material 2. Supplemental Fig. 2. The conditional inference tree of severe PHLF. The branch points stratified participants into 19 nodes according to risk of severe PHLF. Abbreviations: PHLF, post-hepatectomy liver failure. [file 12885_2024_12535_MOESM2_ESM.jpg]
